# The specificity, situational modulations, and behavioral correlates of parent-child neural synchrony

**DOI:** 10.3389/fnhum.2022.1000826

**Published:** 2022-11-09

**Authors:** Yi Liu, Jiaxin Li, Qi Wang, Yarong Li

**Affiliations:** School of Psychology, Northeast Normal University, Changchun, China

**Keywords:** parent-child, neural synchrony, social interaction, social development, fNIRS

## Abstract

In recent years, aiming to uncover the neural mechanism of parent-child interaction and link it to the children’s social development, a newly developed index, namely, parent-child inter-brain neural synchronization (INS) has attracted growing interest. Existing studies have mainly focused on three aspects of the INS; these are the specificity of the INS (i.e., stronger INS for parent-child dyads than stranger-child dyads), the situational modulations of the INS (i.e., how the valence of the situation or the types of interaction modulate INS), and the associations between the INS and the state-like behavioral tendencies or trait-like individual features of the parents and children. This review summarizes the existing findings in line with these three topics and provides preliminary suggestions to promote parent-child INS. In the meanwhile, the inconsistent findings and unstudied questions were discussed, opening new avenues for future studies.

## Introduction

In the past three decades, parent-child synchrony has been used to describe the temporal correlations of the behavior (e.g., gaze), affective states (e.g., positive affect), and biological rhythms (e.g., heart rhythms) between the parent and the child (see Feldman, 2007a for a review). The parent-child synchrony has also been shown to contribute to children’s cognitive, social, and emotional growth (Feldman, 2007a,b,c). More recently, with the fast development of modern neuroimaging techniques (e.g., functional near-infrared spectroscopy, fNIRS and magnetoencephalography, MEG), it is possible to simultaneously record the neural activities of two or more individuals, i.e., hyperscanning, allowing the identification of synchronous features at the neural level when two or more persons are engaged in real-time reciprocal interactions. As a result, a growing number of researchers shift their attention from the single-brain activities toward the study of inter-brain neural synchronization (INS) to uncover the neural mechanisms of social interaction. The INS describes similar patterns of the brain activities across participants at the same time, and it is associated with behavioral synchronization and successful communication (Cui et al., 2012; Jiang et al., 2012). Under the interactive context, the INS reflects the emergent dynamics such as mutual understanding or shared psychological state between interactors, while under non-interactive situations (e.g., watching videos) the INS reflects the shared representations of the dyads during social stimuli processing (Cui et al., 2012; Hasson et al., 2012; Redcay and Schilbach, 2019). In the field of developmental psychology, researchers employ the technique to uncover the neurobiological underpinnings of parent-child interaction. More specifically, researchers use the INS indices to describe the neural similarity of the parent-child dyads during social interaction or social stimuli processing and attempt to associate the parent-child INS with the interactive features of the interaction or the individual’s features of the dyads.

Existing studies have investigated the parent-child INS using fNIRS (Reindl et al., 2018; Azhari et al., 2019, 2020, 2021; Miller et al., 2019; Nguyen et al., 2020a,b, 2021b; Quiñones-Camacho et al., 2020; Wang et al., 2020; Zhao et al., 2021), MEG (Levy et al., 2017), Electroencephalograph (EEG) (Endevelt-Shapira et al., 2021; Deng et al., 2022a,b; Zivan et al., 2022), and Optical topography (OT) (Bembich et al., 2022) to record the brain activities of parent-child dyads simultaneously. The simplest way to calculate the INS is to compute the cross-correlation of the time-series signals between each dyad (Azhari et al., 2020, 2021; Quiñones-Camacho et al., 2020; Zivan et al., 2022). The most used technique is the wavelet transform coherence (WTC) analysis, which estimates a coherence coefficient between two time series of each dyad as a function of frequency and time reflecting both time-related and frequency-related properties of the two time-series (Reindl et al., 2018, 2022; Miller et al., 2019; Nguyen et al., 2020a,b, 2021b; Wang et al., 2020; Kruppa et al., 2021; Zhao et al., 2021; Bembich et al., 2022). Another technique (yet seldomly) used to calculate INS is the dynamic time warping (DTW) time-series analysis, which arranges all sequence points to optimize the alignment of two sequences (Azhari et al., 2019). For MEG or EEG indices, the INS can be calculated as the inter-brain weighted phase lag index (wPLI) reflecting the phase coupling of inter-brain activities from the dyad (Levy et al., 2017; Endevelt-Shapira et al., 2021). In addition, the phase-locking-value (PLV) index measuring whether the EEG signals from the two interacting individuals are phase locked across time was also used as the INS index (Deng et al., 2022a,b). Further, the INS can be defined as time-aligned or time-lagged neural synchronization. For time-aligned INS, the synchronization is calculated using the temporally aligned brain activities of two individuals. Whereas for the time-lagged INS, the brain activity of one individual temporally lags behind that of the other individual, which reflects interpersonal predictive coding and delayed processing during social interaction (Jiang et al., 2021). To our knowledge, only one study used time-lagged WTC as the index of parent-child INS (Zhao et al., 2021), while the remaining studies used time-aligned parent-child INS indices (Levy et al., 2017; Reindl et al., 2018; Azhari et al., 2019, 2020, 2021; Miller et al., 2019; Nguyen et al., 2020a,b, 2021b; Quiñones-Camacho et al., 2020; Wang et al., 2020).

By using these INS indices, existing research focused on three main aspects of the parent-child INS as follows: (i) Specificity of the parent-child INS. This line of research compares the parent-child INS and stranger-child INS in either interactive or non-interactive situations and points out how long-term parent-child attachment shapes the interbrain function between parents and the child. (ii) Modulations of situational factors on the parent-child INS. This line of investigation compares the parent-child INS in different situations (e.g., cooperation or competition) to investigate how the parent-child INS is modulated by the ongoing social cognitive processes. Last but not least, (iii) the behavioral correlates of the parent-child INS. This line of research associates the parent-child INS with the state-like behavioral tendencies or trait-like features of the parent and the child, with the aim to formulate interpretations and identify implications concerning the parent-child INS. The current work will review the parent-infant/child/adolescent hyperscanning research published before October 2022 based on these three lines of investigation (see Table 1). Moreover, we will highlight the progress achieved by these studies and most importantly, we will bring to light critical limitations that can nevertheless be addressed in future studies.

**TABLE 1:** Summary of the literatures about parent-child neural synchrony.

References	Subjects	Techniques	Task	Variables manipulation	INS calculation	Brain regions	Results		
							Specificity	Situational modulations	Behavioral correlates
Azhari et al., 2019	31 Mother-child dyads; Children’s gender: 18 boys and 13 girls; Mother’s age: 34.9 ± 4.16 years; Children’s age: 3.47 ± 0.51 years	fNIRS	Non-interaction (video watching)	Mother-child dyads vs. randomly paired stranger-child dyads	Time aligned; DTW	Left inferior frontal gyrus; frontal eye field; dorsolateral PFC	Mother-child dyads > randomly paired dyads (uncorrected)	Video positivity (null)	Mother-child dyads: • Parenting stress (-)
Azhari et al., 2020	31 Mother-child dyads; Children’s gender: 18 boys and 13 girls; Mother’s age: 34.9 ± 4.16 years; Children’s age: 3.47 ± 0.51 years	fNIRS	Non-interaction (video watching)	Mother-child dyads vs. randomly paired stranger-child dyads	Time aligned; Cross- correlation	Medial PFC	Mother-child dyads = randomly paired stranger-child dyads		Mother-child dyads: • Maternal attachment anxiety (-)
Azhari et al., 2021	29 Father-child dyads; Children’s gender: 18 boys and 11 girls; Father’s age: 38.1 ± 3.67 years; Children’s age: 3.52 ± 0.44 years	fNIRS	Non-interaction (video watching)	Positive scene vs. conflict scene; Father-child dyads vs. randomly paired stranger-child dyads	Time aligned; Cross correlation	Medial left PFC	Conflict scene: father-child dyads > randomly paired dyads	Conflict scene: father-child dyads > randomly paired dyads; Positive scenes: father-child dyads = randomly paired dyads	Father-child dyads: • Father’s age (-)
Bembich et al., 2022	16 Mother–infant dyads; Children’s gender: 8 boys and 8 girls; Mother’s age: 37.84 ± 3.09 years; infants’ age: 2 days	Optical topography	Non-interaction (Watching painful stimulation on infants)	Baseline phase vs. Disinfection phase vs. Painful stimulation	Time aligned; WTC	Mother’s parietal cortex; infants’ motor/somato sensory cortex		Significant INS during painful stimulation	
Deng et al., 2022a	12 Parent-LSA dyads (5 mothers and 7 fathers): Children’s gender: 5 boys and 7 girls; Mother’s age: 40.20 ± 3.49 years; Father’s age: 47.43 ± 4.54 years Children’s age: 11.75 ± 1.48 years; 13 Parent- HSA dyads (7 mothers and 6 fathers): Children’s gender: 9 boys and 4 girls; Mother’s age: 42.49 ± 5.78 years; Father’s age: 42.67 ± 3.50 years; Children’s age: 12.08 ± 1.12 years	EEG	Non-interaction (Watching emotional pictures)	HAS vs. LSA; Positive vs. negative vs. neutral	Time aligned; PLV	Fz, Pz, Cz		Positive: HSA > LSA; Negative: LSA > HSA; LSA: Negative > Neutral; HSA: Positive > Negative	
Deng et al., 2022b	15 LFC parent-adolescent dyads (6 mothers and 9 fathers): Children’s gender: 11 boys and 4 girls; Mother’s age: 43.56 ± 5.43 years; Father’s age: 44.22 ± 4.63 years; Children’s age: 12.00 ± 1.25 years; 14 HFC parent-adolescent dyads (7 mothers and 7 fathers): Children’s gender: 11 boys and 3 girls; Mother’s age: 42.57 ± 1.90 years; Father’s age: 42.76 ± 3.21 years; Children’s age: 12.36 ± 1.08 years	EEG	Non-interaction (video watching)	Positive vs. negative vs. neutral; LFC vs. HFC	Time aligned; PLV	Fz, Pz, Cz		Positive: HFC > LFC; HFC: Positive > Negative	
Endevelt-Shapira et al., 2021	Face-to-face: 62 Mother-infant dyads; Children’s gender: 34 boys and 28 girls; Mothers’ age: 33.3 ± 4.0 years; Children’s age: 7.0 ± 1.49 months; Back-to-back: 39 Mother-infant dyads and 51 stranger-infant dyads: Mother-infant dyads: Children’s gender: 23 boys and 16 girls; Mother’s age: 32.7 ± 4.3 years; Children’s age: 6.8 ± 1.1 months; Stranger-infant dyads: Children’s gender: 26 boys and 25 girls; Stranger age: 34–35 years; Children’s age: 6.8 ± 1.3 months	EEG	Interaction (free interaction)	Mother-child dyads vs. stranger-child dyads; face-to-face interaction vs. back-to-back interaction; With vs. without maternal body odor	Time aligned; wPLI	Central, parietal, and temporal areas	Face-to-face: mother-child dyads > stranger-child dyads; With maternal body odor: Mother-child = stranger-child; Stranger-child dyads: with > without maternal body odor	Mother-child dyads: Face-to-face > back-to-back	Stranger-child dyads with maternal body odor: • Child’s visual attention (+); • Child’s positive arousal (+); • Child’s safety and engagement (null)
Kruppa et al., 2021	Control sample (TD group): 41 Parent-child dyads (40 mother and 1 father) and 32 stranger-child dyads; Children’s gender: 18 boys and 23 girls; Parents’ age: 44.98 ± 5.14 years; Strangers’ age: 23.40 ± 3.72 years; Children’s age: 12.66 ± 2.79 years; Matched sample: 18 Parent-ASD child dyads, 18 Parent-TD child TD dyads and 32 stranger-child dyads; Parent-ASD dyads: Children’s gender: 18 boys; Parents’ age: 46.49 ± 5.57 years; Strangers’ age: 23.40 ± 3.72 years; Children’s age: 13.54 ± 2.96 years; Parent-TD dyads: Children’s gender: 18 boys; Parents’ age: 47.15 ± 4.68 years; Strangers’ age: 23.53 ± 4.40 years; Children’s age: 13.53 ± 2.99 years	fNIRS	Interaction (button press task)	Parent-child dyads vs. Stranger-child dyads vs. randomly paired stranger-child dyads; Cooperation vs. Competition; ASD vs. TD	Time aligned; WTC	DLPFC, FPC	Control sample: Parent-child dyads > Stranger-child dyads (marginally); Parent-child dyads > randomly paired stranger-child dyads; Matched sample: Parent-ASD child dyads = randomly paired stranger-child dyads;	Control sample: Competition > Cooperation	Control sample: • Child’s age (+);
Levy et al., 2017	Mother-child dyads Children’s gender: not reported Mothers’ age: 41.58 ± 4.69 years; Children’s age: 11.67 ± 0.89 years	MEG	Non-interaction (video watching)	Own videos vs. other’s videos; Behavioral synchrony episodes vs. non-synchrony episodes	Time aligned; wPLI (gamma-band phase coupling); correlation coefficients (gamma-band power coupling)	STS		Own videos: phase coupling: behavioral synchronized episodes > behavioral non-synchronized episodes; power coupling: behavioral synchronized episodes = behavioral non-synchronized episodes	
Miller et al., 2019	28 Mother-child dyads; Children’s gender: 13 boys and 15 girls; Mother’s age: 45.93 ± 3.76 years; Children’s age: 11.27 ± 1.27 years	fNIRS	Interaction (button press task)	Cooperation vs. independent task	Time aligned; WTC	Right dorsolateral and frontopolar PFC		Cooperation > independent task	Cooperation: • Children’s anxious attachment (null) • Children’s avoidant attachment (null)
Nguyen et al., 2020a	40 Mother-child dyads; Children’s gender: 20 boys and 20 girls; Mother’s age: 36.37 ± 4.51 years; Children’s age: 5.07 ± 0.04 years	fNIRS	Interaction (free conversation)		Time aligned; WTC	TPJ; dorsolateral PFC			• Amounts of turn-taking (+) • High turn-taking conversation: time course (+)
Nguyen et al., 2020b	42 Mother-child dyads; Children’s gender: 19 boys and 23 girls; Mother’s age: 36.42 ± 4.81 years; Children’s age: 5.08 ± 0.04 years	fNIRS	Interaction (puzzle-solving task)	Cooperation vs. independent task	Time aligned; WTC	TPJ; dorsolateral PFC		Cooperation > independent and rest	Cooperation: • Behavioral reciprocity (+) • Cooperative performance (+) • Maternal stress (-) • Child agency (+) • Children’s temperamental negative affectivity (null) • Maternal sensitivity (null)
Nguyen et al., 2021a	72 Mother-infant dyads; Children’s gender: 39 boys and 33 girls; Mothers’ age: 33.97 ± 4.94 years; Children’s age: 4.7 months ± 16 days	fNIRS	Non-interaction (video watching); Interaction (free play)	Proximal joint watching vs. distal joint watching vs. free play (face-to-face interaction)	Time aligned; WTC	Lateral PFC; Medial PFC		Free play > proximal joint watching > distal joint watching	• Affectionate touch (+) • Stimulating touch (-) • Passive touch (null) • Functional touch (null) • Interpersonal physiological synchronization (null)
Nguyen et al., 2021b	66 Father-child dyads; Children’s gender: 35 boys and 31 girls; Father’s age: 339.2 ± 5.17 years; Children’s age: 5.32 ± 0.31 years	fNIRS	Interaction (puzzle-solving task)	Cooperation vs. independent task	Time aligned; WTC	Bilateral dorsolateral PFC; left TPJ		Cooperation > independent and rest	Cooperation: • Father’s role attitude (+) • Behavioral reciprocity (null) • Cooperative performance (null) • Paternal sensitivity (null) • Paternal stress (-, marginal) • Child agency (null)
Quiñones-Camacho et al., 2020	116 Mother-child dyads; Children’s gender: 63 boys and 53 girls; Mother’s age: not reported; Children’s age: 4.86 ± 0.60 years	fNIRS	Interaction (puzzle-solving task)	Frustration phase vs. recovery phase	Time aligned; Robust correlation coefficient	Lateral PFC		Frustration = recovery	• Frustration: Behavioral synchrony (+) • Recovery: Behavioral synchrony (null) • Child irritability (-) • Maternal irritability (null)
Reindl et al., 2018	30 Mother-child dyads and 3 father-child dyads; 21 stranger-child dyads; Children’s gender: 18 boys and 15 girls; Parents’ age: 41.24 ± 4.32 years; Strangers’ age: 24.33 ± 4.70 years; Children’s age: 7.52 ± 0.87 years	fNIRS	Interaction (button press task)	Parent-child dyads vs. stranger-child dyads; Cooperation vs. competition	Time aligned; WTC	left dorsolateral and frontopolar PFC	Cooperation: parent-child > stranger-child	Parent-child: cooperation > competition	Parent-child cooperation: • Parent’s and child’s emotion regulation ability (+) • Cooperative performance (+)
Reindl et al., 2022	34 mother-child dyads and 29 stranger-child dyads: Children’s gender: 9 boys and 4 girls; Mother’s age: 45.32 ± 4.95 years; Stranger’s age: 23.07 ± 2.09 years; Children’s age: 14.26 ± 2.21 years	fNIRS	Interaction (button press task)	Parent-child dyads vs. Stranger-child dyads; Cooperation vs. Competition	Time aligned; WTC	Prefrontal cortex	Mother-child dyads > Stranger-child dyads		Competition: • Autonomic nervous system synchrony (+); • Behavioral synchronous responses (-)
Wang et al., 2020	12 Mother-child dyads and 4 father-child dyads; Children’s gender: 15 boys and 1 girls; Parents’ age: not reported; Children’s age: 8.2 ± 1.7 years	fNIRS	Interaction (button press task)	Cooperation vs. independent task	Time aligned; WTC	Superior frontal cortex		Cooperation > independent task	Cooperation-independent: • Autism symptoms (-) • Cooperative performance (+)
Zhao et al., 2021	25 Mother-child dyads and stranger-child dyads; Children’s gender: 13 boys and 12 girls; Mother’s age: 37.84 ± 3.09 years; Stranger’s age: 37.12 ± 4.48 years; Children’s age: 7.40 ± 0.28 years	fNIRS	Interaction (cooperative drawing)	Mother-child dyads vs. stranger-child dyads	Time lagged (4s); WTC	Left TPJ			Mother-child dyads: • Children’s committed compliance (+) • Children’s responsiveness (+) • Mutual responsiveness (-) • Mother’s responsiveness (null)
Zivan et al., 2022	24 Mother-child dyads: Children’s gender: 16 boys and 8 girls; Mother’s age: 35 ± 5 years; Children’s age: 33.5 ± 5.8 months	EEG	Interaction (Dialogic Reading)	Uninterrupted vs. Interrupted; Mother-child dyads vs. randomly paired dyads	Time aligned; Circular Correlation	Mother’s left and child’s right hemispheres	Mother-child dyads > randomly paired dyads	Uninterrupted > Interrupted	Uninterrupted: • Child’s age (+); • Mother reading skills (+); • Mother’s passive screen exposure (-); Uninterrupted vs. interrupted: • Joint attention (+)

fNIRS, functional near-infrared spectroscopy; MEG, magnetoencephalography; WTC (), wavelet transform coherence (frequency hand); DTW, dynamic time warping; wPLI, weighted phase lag index; PLV, phase-locking-value; STS, superior temporal sulcus; PFC, prefrontal cortex; TPJ, temporo-parietal junction; TD, typically developing; ASD, autism spectrum disorder; LSA, low social anxiety; HSA, high social anxiety; LFC, low family cohesion; HFC, high family cohesion; (+)/(-)/(null), positive/negative/no correlation between the behavioral indices with INS; >, the INS in the former condition was stronger than that in the later condition. = : The INS in the former condition was comparable with that in the later condition. The behavioral correlates results only listed the behavioral indices that associated with the INS indices, while that associated with brain activations was not listed.

## Specificity of the parent-child inter-brain neural synchronization

The specificity of the parent-child INS, namely, the stronger INS of parent-child dyads than stranger-child dyads, was found in both non-interactive (e.g., watching videos without communication) and interactive situations (e.g., cooperative problem solving).

In non-interactive situations, parent-child dyads passively watched videos together without explicit cognitive tasks. Since the contents of the videos were consistent across all participants, the INS of parent-child dyads was compared with that of randomly paired stranger-child dyads (i.e., in data analysis, each child was randomly paired with a parent of another child to calculate the stranger-child INS as control). We regard the stronger parent-child INS (compared to INS of randomly paired stranger-child) as the specificity of the parent-child INS. For example, Azhari et al. used fNIRS to record the INS of mother-child dyads (Azhari et al., 2019) and father-child dyads (Azhari et al., 2021) while watching animated video clips (including scenes of positive family interactions and emotionally arousing family conflict). Compared with randomly paired stranger-child dyads, father-child dyads showed stronger INS in left frontal regions while watching family conflict scenes, moreover, the specificity of father-child INS was more obvious for younger fathers (Azhari et al., 2021). Somewhat similar, the specificity of mother-child INS in the left frontal region while video watching was also significant (under *p* < 0.05 level but did not survive multiple comparisons correction), but only when analyzing the data of both positive and conflict scenes together (Azhari et al., 2019). An exception was observed in Azhari et al. (2020), in which the INS of mother-child dyads was comparable with randomly paired stranger-child dyads. The authors concluded that concurrent experiences in the laboratory (e.g., watching the same video) induced high levels of INS even for randomly paired dyads which in turn overruled the specificity of parent-child INS normally derived from specific relationship attachment. In general, these results demonstrate a tendency of parent-child INS to be stronger than randomly paired stranger-child INS under non-interactive situations. Nevertheless, there are other important factors that could interact with effect and should be taken into consideration, such as the gender and age of the parent and valence of the scenes.

In interactive situations, the control condition was set as stranger-child dyads under the same interactive tasks as parent-child dyads. The specificity of parent-child INS was indexed by the stronger INS of parent-child dyads compared to that of interactive stranger-child dyads. Endevelt-Shapira et al. (2021) showed that the parent-infant (about 7-month-old) INS was stronger than stranger-infant INS during face-to-face interaction, and the exposure to maternal chemosignals during stranger-infant interaction attenuated this difference. For children about 8-years-old, Reindl et al. (2018) asked parent-child dyads and stranger-child dyads to play a computer game cooperatively (pressing a button as simultaneously as possible to win the game) or competitively (pressing a button as soon as possible to beat the other player). Stronger INS was found in the left dorsolateral prefrontal and frontopolar cortex for the parent-child dyads in comparison to the stranger-child dyads during cooperation. With the same task, Reindl et al. (2022) replicated the stronger INS for parent-child dyads than stranger-child dyads during cooperation as well as competition. While for typically developing adolescents (8–18 years) in Kruppa et al. (2021), the difference between parent-adolescent and stranger-adolescent (actual dyads) INS was marginally significant for HbR signals, and the difference between the parent-adolescent dyads and randomly paired dyads was significant during competition as well as cooperation. However, the increased parent-adolescent INS was not found for adolescents with autism spectrum disorder. These results demonstrated a trend of the specificity of parent-child INS in interactive situations, especially for typically developing children.

Taken together, these results demonstrate that the specificity of the parent-child INS exists both in the interactive and non-interactive situations. The main explanation is based on the specific parent-child attachment. That is, the parent-child attachment that formed through long-term parenting results in stronger affective bonding and more joint experiences than that of non-parent relationships. Hence, the non-interactive situations such as watching videos together, would induce more joint processing or shared representations of the social scenes for parent-child dyads, thus leading to stronger INS compared to randomly paired stranger-child dyads (Azhari et al., 2021). Similarly, in the interactive situations parent-child dyads could better understand each other (compared to stranger-child dyads) during cooperation (Reindl et al., 2018) and could engaged in strong social comparison process during competition (Reindl et al., 2018; Kruppa et al., 2021) which resulted in stronger parent-child INS. Such conclusions, however, must be interpreted with caution. For instance, the explanations regarding the effect of attachment on the parent-child INS are rather speculative and lack empirical evidence. What is more, the potential of genetic effect on the specific parent-child INS cannot not be ignored, although this line of investigation remains largely untapped. Finally, the present review demonstrates that there are critical limitations on the specificity of the parent-child INS, especially regarding cooperative or competition situations (Reindl et al., 2018; Kruppa et al., 2021), processing conflict narrative scenes (Azhari et al., 2021), and the gender and age of the parent (Azhari et al., 2020, 2021; Kruppa et al., 2021). However, these limitations have been interpreted from scattered findings and were not tested directly.

## Situational modulations on the parent-child inter-brain neural synchronization

For infants as early as the second postnatal day, the mother’s left parietal cortex activity synchronized to her newborn’s brain activity in superior motor/somatosensory cortex when the newborn was in potential danger situation (painful stimulation) (Bembich et al., 2022). Later, namely, 4–8 months, the mother-infant INS during face-to-face interaction was stronger than that during non-interactive situations (Endevelt-Shapira et al., 2021; Nguyen et al., 2021a), and in the non-interactive situations, the mother-infant INS was stronger during proximal than distal joint video watching (Nguyen et al., 2021a). These findings suggest that the parent-child INS occurs early in life and highly depends on the interactive situations.

For children (>2 years) participants, the situational modulations on the parent-child INS were tested in interactive and non-interactive situations.

In non-interactive situations, the main modulator is the valence of the videos/pictures watched by the parent-child dyads. Levy et al. (2017) videotaped a positive dialog (i.e., planning a fun day) and a conflict dialog (a discussion regarding a conflict that happened before the dialog took place) between mother and child at home. The videos of the dialogs were encoded as behavioral synchronized episodes (moments when mother and child expressed simultaneous positive affect) and non-synchronized episodes (neither mother nor child expressed positive affect). The mother-child dyads’ brain activities were recorded using MEG while they watched videos of themselves and of other mother-child dyads. The results showed that the parent-child gamma-band phase coupling in superior temporal sulcus (STS) was significant while watching their own synchronized episodes. Given that the behavioral synchrony was defined by the mother and child simultaneous expression of positive affect, while non-synchrony was defined by the lack of positive affect from both parts, from the perspective of situational valence, these results indicated that positive situations during social interactions may promote the mother-child INS. However, Azhari et al. (2021) found that the stronger INS of father-child dyads (vs. randomly paired stranger-child dyads) in the frontal regions was only observed while watching videos of family conflict scenes but not during the positive scenes, indicating that the father-child INS was more likely to be induced by situations of negative valence. The authors explained this result as that negative scenes induce higher emotional arousal compared to positive scenes, thus requiring individuals to make greater effort to mentalize the other’s emotional states, which finally produces higher INS in the social brain regions. In addition, Azhari et al. (2019) reported null correlations between the mother-child INS and video positivity. Whereas in a later study, Azhari et al. (2020) distinguished the valence of the video stimuli but did not report the difference of the mother-child INS between positive and negative situations. With adolescents as participants (10–14 years), Deng et al., 2022a,b asked parent-adolescent dyads to watch emotional pictures or videos during EEG recording. The parent-adolescent INS (PLV in gamma band) was stronger during watching positive stimuli than negative stimuli for high social anxiety adolescents and their parents (Deng et al., 2022a) and for the dyads from high family cohesion (i.e., emotion, support, helpfulness, and caring among family members) (Deng et al., 2022b). In contrast, the INS in negative condition was stronger than that in positive condition for low social anxiety adolescents (Deng et al., 2022a). The dyads from low family cohesion showed comparable INS for positive and negative conditions (Deng et al., 2022b). Overall, researchers have already realized that in non-interactive situations, the valence of the situation affects parent-child INS. Nonetheless, the results were inconsistent and future research on this question will strengthen this idea by considering the individual and family features of the dyads such as gender of the parent, age of the child, social traits and family functioning.

In interactive situations, Zivan et al. (2022) showed that continuous storytelling interaction induced stronger parent-child INS than interrupted situations, in which the parent answered the messages on the mobile phone during the storytelling, suggesting that the continuity of the interaction was important for INS. Besides this, the main modulators that have been taken into account are the ways in which the parent-child dyads interact, i.e., cooperation, competition, or perform the task independently. Reindl et al. (2018) showed that when compared with competition (pressing a button as soon as possible), cooperation (pressing a button as simultaneously as possible) induced stronger parent-child INS in the dorsolateral prefrontal and frontopolar cortex. Similarly, Miller et al. (2019) used an independent task in which the mother and the child completed a (computer) reaction time task independently as the control condition and found that cooperation induced stronger mother-child INS in the right dorsolateral and frontopolar prefrontal cortex. A stronger parent-child INS in the frontal cortex during cooperation (vs. independent condition) in the reaction time task was also found for children with autism and their parents (Wang et al., 2020). In addition, in a tangram puzzle-solving task (arranging seven geometric shapes to recreate different templates), similar results were found for mother-child (Nguyen et al., 2020b) and father-child (Nguyen et al., 2021b) dyads. That is, compared with independent problem solving or rest (no task), cooperative problem solving induced stronger parent-child INS in the bilateral prefrontal cortex and temporo-parietal areas. Further, using the same cooperative puzzle-solving task, Quiñones-Camacho et al. (2020) distinguished the frustration phase (solving difficult puzzles that induced negative affect on the child) and the following recovery phase (freely playing with toys to recover from the negative affect) and showed comparable mother-child INS in these two phases. One exception was Kruppa et al. (2021), in which stronger parent-adolescent INS was found in competition than cooperation for typically developing groups. But for adolescents with autism spectrum disorder, the INS during competition and cooperation were comparable. Of note, they used adolescents as participants (8–18 years). The development of parent-adolescent relationships and conflict interactions were raised as potential explanations for the high INS during competition. Overall, exclusive of adolescents, the results presented herein consistently showed that cooperative interactions of the parent and the child induced stronger INS in the social brain regions (e.g., frontal cortex, temporo-parietal areas, and STS) compared to competitive interactions, no interactions and rest. The cooperation advantage for parent-child INS was consistently explained in terms of the situational features of the cooperation. That is, compared with competition and independent tasks, cooperation induces more joint attention and similar emotional reactions, and needs continuous adjustment of the behaviors to achieve the goals of cooperation, which requires more effort to understand each other’s intentions and mental states. These similar social cognitive processes induced the high levels of parent-child INS during cooperative interactions (Reindl et al., 2018; Miller et al., 2019; Nguyen et al., 2020b,2021b; Wang et al., 2020).

Taken together, there is no conclusive evidence that the valence of a situation modulates parent-child INS in non-interactive situations. Whereas in interactive situations, findings frequently show that cooperation (compared to competition or independent tasks) induces stronger parent-child INS possibly because of joint attention, shared emotion, mentalizing and behavioral adjustment during cooperation.

## Behavioral correlates of the parent-child inter-brain neural synchronization

In the field of developmental psychology, how the parent-child INS correlates with the parents’ and children’s social behaviors or traits are of great significance to understand the development of the children’s social cognition and social brain. Previous studies have focused on two types of behavioral indices that correlate with parent-child INS, namely, state-like, and trait-like indices. The former is highly dependent on current situations and is often coded during ongoing social interactions, such as communicative reciprocity and cooperative performance. The latter relates to inherent traits or behavioral tendencies that are consistent across time and independent of the ongoing situations, such as the parents’ age and children’s irritability.

### State-like indices

In previous studies, the most mentioned state-like indices that correlate with parent-child INS are the interactive features during interaction and the consequence of the interaction (e.g., cooperative performance). Interactive features such as affective touch during parent-infant free playing was positively correlated with INS, while stimulating touch was negatively correlated with INS (Nguyen et al., 2021a). Communicative reciprocity (i.e., verbal turn-taking) during free conversation between the mother and child positively predicted mother-child INS in frontal and temporoparietal areas, while the INS also increased over the course of the conversation (Nguyen et al., 2020a). The behavioral reciprocity (i.e., contingent responses) during the puzzle-solving cooperation was positively correlated with mother-child INS in the bilateral prefrontal cortex and temporo-parietal areas (Nguyen et al., 2020b) but not with father-child INS (Nguyen et al., 2021b). During computer game competition, less behavioral synchronous responses were associated with higher INS in Reindl et al. (2022). However, Kruppa et al. (2021) did not find significant correlations between motor synchrony (differential response times of each dyad) and the INS. Using gaze features as the measurement of joint attention, Zivan et al. (2022) showed a positive association between the parent-child INS and joint attention during storytelling. Apart from these reciprocal features, child agency (i.e., interest, vigor, enthusiasm, and eagerness to do the tasks) during cooperation tended to be positively correlated with mother-child INS (Nguyen et al., 2020b) but not with father-child INS (Nguyen et al., 2021b). Whereas parent’s sensitivity (i.e., prompt, appropriate, and sensitive responses to the child’s signals) during cooperation did not correlate with the father-child (Nguyen et al., 2021b) or the mother-child (Nguyen et al., 2020b) INS. The explanation offered for these positive associations between interactive features and INS was that, the high quality of interaction meant behavioral coordination or synchronization with the signs of joint attention, shared emotions or social adaptive motivation thus promoting INS (Nguyen et al., 2020a,2021b,Zivan et al., 2022).

However, it should be noted that the positive association is not always observed for father-child dyads (Nguyen et al., 2021b), or on the contrary, it is even shown to be negative (Zhao et al., 2021). Zhao et al. (2021) used a “mother-guided” cooperative drawing task and coded the child’s, mother’s and mutual responsiveness and the child’s committed compliance during drawing. The results showed that the mother-child time-lagged INS in the left temporoparietal junction was positively correlated with the child’s responsiveness and committed compliance behavior but not with the responsiveness of the mother. In contrast, the mother-child time-lagged INS was negatively correlated with the mutual responsiveness and the child’s committed compliance behavior, which is inconsistent with the results of Nguyen et al., 2020a,b, 2021b. The inconsistency may be a result of the different INS indices. Zhao et al. (2021) used time-lagged (i.e., the child’s brain activity lagged behind that of the mother by 4 s) INS, while other studies used time-aligned INS (Nguyen et al., 2020a,b, 2021b). The drawing task in Zhao et al. (2021) was easy for the mother but relatively difficult for the child, and the mother had to teach and guide the child in the drawing task. Thus, the 4 s time lag might reflect a delayed processing of the mother’s beliefs in the child’ mind during interactions. The inconsistency observed in these results might suggest that mutual communication benefits parent-child INS only for cooperation between equally contributed dyads but not for the leader-follower dyads. Apart from the different INS indices (time-aligned or time lagged), it is also worth noting that some of these studies tested mother-child dyads and some tested father-child dyads and these studies used different cooperative tasks (free conversation, puzzle-solving, drawing, etc.). These different experiment settings restrict the comparability of results and may host potential modulators on the parent-child INS, such as task difficulty, and parent gender.

Apart from the interactive features during interaction, the final cooperative performance also received great attention from investigators. For example, Nguyen et al. (2020b) found that stronger mother-child INS in the bilateral prefrontal cortex and temporo-parietal areas during puzzle-solving cooperation was positively correlated with the cooperative performance (number of templates solved). Similar results were also found for children with autism spectrum disorder, where the stronger the parent-child INS in the frontal cortex was, the better was their cooperative performance (simultaneous button press) (Wang et al., 2020). What is more, Reindl et al. (2018) tested the causal relation between the parent-child INS and cooperative performance. They divided the cooperation processes (button press task) into two blocks and found that, the parent-child INS in block 1 positively predicted the cooperative performance in block 2, while the cooperation performance in block 1 did not predict the parent-child INS in block 2. This result suggested that the INS could yield better cooperative performance while the cooperative performance did not affect the INS. Note that in the above-mentioned studies, all the participants (Nguyen et al., 2020b) or most of them (Reindl et al., 2018; Wang et al., 2020) were mother-child dyads (Nguyen et al., 2020b). On the contrary, when using father-child dyads as participants, null correlation between the cooperative performance and the INS during puzzle-solving cooperation (vs. independent condition) was found, suggesting that the neural dynamics in father–child problem solving can diverge from mother–child problem solving (Nguyen et al., 2021b). To sum up, most of the existing evidence supported a positive association between parent-child INS (especially for mother-child INS) and cooperative performance (Reindl et al., 2018; Nguyen et al., 2020b; Wang et al., 2020). The explanation is that the INS during cooperation facilitates exchanges of social information between the dyad which helps to achieve the cooperation goal. These findings indicate that the INS may serve as a neural mechanism of successful social cooperation (Reindl et al., 2018; Nguyen et al., 2020b), while the low level of INS may also underlie the social deficits of children with autism disorder (Wang et al., 2020).

Taken together, the association between interactive features and parent-child INS was not consistently observed. In contrast, findings that higher levels of parent-child INS led to better cooperative performance were relatively robust, suggesting a brain-to-brain neural mechanism of successful parent-child cooperation.

### Trait-like indices

The trait-like indices of the parent and the child have both been shown to be correlated with parent-child INS. The most mentioned parents’ trait-like indices are the emotion-related indices. For example, mother’s greater parenting stress was associated with weaker mother-child INS in the medial prefrontal cortex during non-interactive video watching (Azhari et al., 2019). The higher the general stress (including but not limited to economic stress, and interpersonal stress) the mother perceived, the weaker the mother-child INS in the frontal cortex and temporal-parietal areas was during cooperation (Nguyen et al., 2020b). In the same vein, mother-child INS in the medial prefrontal cortex while watching a video reduced as the maternal attachment anxiety increased (Azhari et al., 2020). Findings concerning the negative associations between stress or anxiety and mother-child INS were explained in terms of the effect of stress or anxiety on the social cognitive process. That is, strong feelings of stress or anxiety may interrupt emotion sharing with the child and prevent the mother from taking the child’s perspective, which result in a low level of mother-child INS (Azhari et al., 2019, 2020; Nguyen et al., 2020b). Nonetheless, the negative association between the father’s perceived parenting stress and father-child INS was relatively weak (marginally significant effects) (Nguyen et al., 2021b). Furthermore, a study by Reindl et al. (2018) focused on the association of parents’ and child’s emotion regulation and the parent-child INS. A mediation model was built and demonstrated that the parents used reappraisal as a strategy to regulate their emotions thus promoting the emotion regulation abilities of the child through increasing the parent-child INS in the dorsolateral prefrontal and frontopolar cortex during cooperation. This finding suggests that the parent-child INS might potentially be one mechanism through which the parent’s emotion regulation influences the child’s emotional development. In addition, the father-child INS in the frontal cortex while watching a video decreased with the father’s age. One possible reason is older fathers processed narrative scenes in the presence of their children without feeling the need to excessively attune to the child’s emotional state, which in turn decreased the father-child INS (Azhari et al., 2021). Nguyen et al. (2021b) found that when the father’s attitude toward their role in child-rearing was more involved, sensitive, and positive, the father-child INS was stronger in the left TPJ and bilateral dorsolateral prefrontal areas during cooperative problem solving. The explanation was that the identification with being a warm and supporting father promoted a higher quality of interaction with their child and resulted in a high level of father-child INS.

Child related trait-like indices include temperament and attachment related indices. However, no evidence was found for the significant associations between the mother-child INS and the negative affectivity of temperament (Nguyen et al., 2020b), or the child’s anxious or avoidant attachment (Miller et al., 2019) during cooperation. Distinguishing the frustration (difficult puzzle-solving) and recovery (free playing) phases, the children’s irritability was not associated with the mother-child INS in frustration phase, while in the following recovery phase the children’s irritability was negatively correlated with the mother-child INS (Quiñones-Camacho et al., 2020). The parent-child INS during storytelling increased with the child’s age (Zivan et al., 2022). Additionally, the children with severe autism symptoms (measured by the communication and imagination subscales of the Autism Spectrum Quotient) showed lower levels of parent-child INS in the frontal regions during cooperation (Wang et al., 2020).

Generally, the trait-like indices includes parents’ stress (Azhari et al., 2019; Nguyen et al., 2020b,2021b), the father’s age (Azhari et al., 2021) and attitude toward the parenting role (Nguyen et al., 2021b), the mother’s and child’s attachment styles (Miller et al., 2019; Azhari et al., 2020), the temperament related features (Nguyen et al., 2020b; Quiñones-Camacho et al., 2020), emotion regulation of the parents and child (Reindl et al., 2018), and the children’s autism symptom (Wang et al., 2020). Among which, the mother’s stress (Azhari et al., 2019; Nguyen et al., 2020b), father’s age (Azhari et al., 2021) and parenting role attitude (Nguyen et al., 2021b), child’s age (Zivan et al., 2022) and temperamental irritability (Quiñones-Camacho et al., 2020), parents’ and child’s emotion regulation (Reindl et al., 2018), and the children’s autism symptom (Wang et al., 2020) showed associations with the parent-child INS in the frontal regions and temporal-parietal areas, while the remaining indices did not show clear associations with parent-child INS. It is worth noting that each trait-like index listed above was mentioned in only one or two studies, and were tested under different tasks (e.g., video watching, cooperation, etc.). Thus, the associations between trait-like indices and parent-child INS should be considered with caution and verified with future studies.

To conclude, the results regarding associations between behavioral indices and parent-child INS remain somewhat unclear. In spite of this, the explanations about the association results were relatively consistent and focused on the association between the behavioral features of the dyad and the brain functions during social interaction. Specifically, the associations between parent-child INS and the state-like indices (e.g., turn-taking and cooperative performance) were explained in terms of the high quality of parent-child interaction reflecting the coordination or synchronization of their behaviors, which facilitated INS (Nguyen et al., 2020a,2021b). As a result, the INS could also promote the social exchanges of the dyad and increase their behavioral performance (Reindl et al., 2018; Nguyen et al., 2020b). In other words, effective social interaction provided the possibility of INS, whereas the high level of INS served as the neural bases for the following effective social interaction (e.g., cooperation). Notably, this explanation emphasized that the INS highly relied on the ongoing social situations, thus it could also explain why the INS showed relatively weak associations with trait-like indices which are independent of the ongoing situations. For example, the attachment style is an important index highly related with parent-child relationships but was not related with parent-child INS during specific social interactive situations (Miller et al., 2019; Azhari et al., 2020). One possible explanation for the null association may be that, the measurement of the attachment reflected long-term parent-child behavioral tendencies, therefore it is difficult to show the effects of attachment styles on parent-child INS in temporary social interactive situations manipulated in the laboratory setting (Azhari et al., 2020; Nguyen et al., 2020b).

## Discussion

### Psychological perspectives

As reviewed above, up to now, the relatively consistent findings regarding parent-child INS depart from two main aspects, namely, the specificity of the parent-child INS (i.e., stronger INS for parent-child dyads than stranger-child dyads) and the situational dependency of the parent-child INS (i.e., the stronger parent-child INS during cooperation than competition or independent situations, and the positive association between parent-child INS and cooperative performance). In contrast, the associations between the parents’/child’s trait-like behavioral indices and INS were complex and did not allow for clear conclusions. Nonetheless, two main aspects influencing the INS could be observed, namely, interpersonal relationship (i.e., parent-child vs. stranger-child) and interactive features (i.e., cooperation vs. competition). Other factors, however, such as individual features (e.g., attachment styles, etc.) of the parents or the children did not show clear influence on the parent-child INS. To recap, interpersonal relationship was the main psychological explanation regarding the specificity of the parent-child INS. That is, the long-term attachment formed between parents and their children marks a specific affective connection with shared experiences and memories, which induce similar neural responses when experiencing the same social events, that ultimately result in high level of parent-child INS (Reindl et al., 2018; Azhari et al., 2021). In contrast, the situational dependency of the parent-child INS was explained in term of the interactive features of the ongoing social interactions such as the shared attention, similar emotional responses, strong intention to understand the other and the mutual adaptability to achieve the cooperative goals (Reindl et al., 2018; Miller et al., 2019; Nguyen et al., 2020b,2021b; Quiñones-Camacho et al., 2020; Wang et al., 2020). Although the influence of the individual features on the parent-child INS were complex, the explanations were also mostly focused on the social cognition processes. For example, some traits effectively facilitated mentalizing or emotion sharing, which affected the INS (Reindl et al., 2018; Azhari et al., 2019, 2020, 2021; Miller et al., 2019; Nguyen et al., 2020b,2021b; Quiñones-Camacho et al., 2020; Wang et al., 2020).

To sum up, two main explanations for the parent-child INS were observed in this review, i.e., long-term formed attachment relationship and the effective social cognitive processes based on the interactive features. These two explanations are complementary to each other. Nevertheless, these explanations were all *post-hoc* inferences and, therefore they lack empirical evidence. For the attachment account, previous studies did not find clear associations between the attachment styles and parent-child INS (Miller et al., 2019; Azhari et al., 2020), while associations between parent-child INS and other relevant indices, such as parent-child relationship, companionship, and co-living time, were not mentioned so far. In addition, the hereditary account for the specificity of the parent-child INS was surprisingly ignored. For the ongoing social cognition explanation, the frequently mentioned emotion sharing and mentalizing processes that depend on the situations were not directly measured in previous studies to provide objective evidence.

### Neuroscience perspectives

The above mentioned psychological explanations of INS are based on the psychological perspective of social interaction. On the other hand, neuroscience findings of the brain functions also support these explanations. Existing studies about the specificity, situational modulation and the behavioral correlates of the parent-child INS typically showed on the frontal cortex (Reindl et al., 2018; Azhari et al., 2019, 2020, 2021; Miller et al., 2019; Nguyen et al., 2020a,b, 2021b; Quiñones-Camacho et al., 2020; Wang et al., 2020), temporal-parietal junction (Nguyen et al., 2020a,b, 2021b; Zhao et al., 2021), and superior temporal sulcus (Levy et al., 2017). These are typical regions of the social brain network, which responsible for mentalizing, emotional control, and attention to social information (Brothers, 1990; Frith and Frith, 2007; Blakemore, 2008). Based on the functions of these brain regions, the parent-child INS in the dorsolateral prefrontal cortex was suggested to represent similar cognitive control processes of the dyad, especially the emotional control process (Reindl et al., 2018). The INS in the medial prefrontal cortex, frontopolar and the temporal-parietal regions were suggested to represent the similar processing of the social information or mentalizing of the dyad (Reindl et al., 2018; Miller et al., 2019; Azhari et al., 2020; Nguyen et al., 2020a,b, 2021b). The parent-child INS in the superior temporal sulcus was suggested to represent the integrations of the bottom-up and top-down processes to create online bio-behavioral coordination between the dyad (Levy et al., 2017).

Concerning these explanations, three points need to be noted. (i) The explanations were drawn from *post-hoc* inferences based on the general function of these social brain regions rather than empirical evidence through strict manipulation or measurement of the social cognition process (e.g., mentalizing or emotional control). Since one brain region would be engaged in multiple cognitive functions, the *post-hoc* functional inferences based on the brain functions should be regarded with caution. (ii) Although the existing studies consistently focused on the social brain regions such as frontal and temporal-parietal regions, the parent-child INS was found in different regions in different studies. The specificity of parent-child INS was consistently found in the frontal regions (Reindl et al., 2018; Azhari et al., 2021). The situational modulations were widely found in the frontal, temporal-parietal junction or superior temporal sulcus (Reindl et al., 2018; Miller et al., 2019; Nguyen et al., 2020b,2021b; Wang et al., 2020). The INS showed significant associations with behavioral indices in the frontal regions or temporal-parietal regions (Reindl et al., 2018; Nguyen et al., 2020a,b, 2021b; Quiñones-Camacho et al., 2020; Wang et al., 2020; Zhao et al., 2021). In addition, these regions showed different hemisphere asymmetry in different studies (Reindl et al., 2018; Azhari et al., 2019, 2020, 2021; Miller et al., 2019; Wang et al., 2020; Zhao et al., 2021), however, these differences on the locations of the parent-child INS were not discussed. (iii) Most of the existing studies used the fNIRS to record the brain activities (Reindl et al., 2018; Azhari et al., 2019, 2020, 2021; Miller et al., 2019; Nguyen et al., 2020a,b, 2021b; Quiñones-Camacho et al., 2020; Wang et al., 2020; Zhao et al., 2021). Because of the limitations posed by fNIRS, only the brain activities of the cerebral cortex regions could be recorded. The parent-child INS in other key nodes in the social brain network such as insular, medial prefrontal cortex, cingulate cortex and precuneus (Brothers, 1990; Frith and Frith, 2007; Blakemore, 2008) have not been investigated yet.

### Inspirations

Generally, the current findings reviewed in the current article indicate that parent-child INS have positive meanings on the children’s social and affective development. For example, high level of parent-child INS reflected high quality of parent-child interaction, facilitated cooperation (Reindl et al., 2018; Nguyen et al., 2020b; Wang et al., 2020) and correlates with child’s committed compliance (Zhao et al., 2021). The parents’ emotional regulation strategies could also promote the development of the child’s emotion regulation abilities through parent-child INS (Reindl et al., 2018). Thus, it can be seen that creating favorable conditions to promote parent-child INS may benefit children’s development.

The existing findings may provide valuable information for the parents as to how to promote parent-child INS. First, existing studies consistently showed that cooperative tasks (relative to competition or independent task) induced higher parent-child INS in the social brain regions possibly through motivating joint attention, inducing similar emotional responses, and promoting understanding and inferring the other’s mental states (Reindl et al., 2018; Miller et al., 2019; Nguyen et al., 2020b,2021b; Wang et al., 2020). Thus, creating cooperative situations in family life may help to produce parent-child INS that benefit the social development of the children. Second, high levels of parent-child INS can be achieved when parents provide appropriate, and in-time responses during parent-child interaction because high quality of social communication and information exchange, such as joint attention, similar emotional responses and motivation to adapt to each other, produces high levels of INS (Nguyen et al., 2020b,2021b). Notably, this conclusion builds on the equal cooperation of the parent and the child. When the cooperation task was too difficult for the child and the parent’s guidance was needed, mutual responsiveness might decrease the parent-child INS (Zhao et al., 2021). Third, parent-child INS could potentially be improved when parents maintain positive psychological states. There is evidence that high levels of stress or anxious attachment prevents the mother from taking the child’s perspective and sharing their emotion, which makes INS hard to achieve (Azhari et al., 2019, 2020; Nguyen et al., 2020b). What is more, parents’ effective emotion regulation could promote the development of the child’s emotion regulation ability through parent-child INS (Reindl et al., 2018). Thus, by keeping a positive psychological state (e.g., good stress coping and emotion regulation) parents can promote the parent-child INS and generate a positive effect on the children’s social and emotional development. Last but not least, the INS can serve as a potential index for evaluating social dysfunctions such as those observed in autistic population. As found in Wang et al. (2020), for children with autism spectrum disorder, the weaker the parent-child INS, the severer the autism symptoms, and the worse the cooperative performance. These findings suggest that the abnormality of INS might underlie the social dysfunctions of autism. Thus, using the INS as a potential biological index of social deficits may be a promising means for the diagnosis and intervention of autism.

Although these benefits from parent-child INS require targeted investigation, the findings aforementioned provided meaningful inspirations and primary guidance for parent-child relationship and family education, which will benefit the child’s social development.

## Limitations and future directions

Overall, the existing research on parent-child INS has made great progress and set the path for the investigation of children’s social development from the perspective of social neuroscience. In spite of this, there are some limitations and unanswered questions that need to be investigated in future studies.

First, the existing findings were preliminary and the explanations of these findings need further empirical evidence. It can be seen that there were some inconsistent findings, such as whether the valence of a situation modulates parent-child INS in non-interactive situations, or which interactive or individual features were associated with the parent-child INS, need to be clarified in future studies. Additionally, some of the findings were only evident in one or two studies (e.g., the associations between trait-like indices and parent-child INS) and would benefit from further replications. Moreover, as mentioned above, the explanations about the parent-child INS (i.e., attachment relationship and social cognitive processes) are mostly speculative and lack empirical evidence, and alternative explanations, such as heredity of the brain functions, need to be further studied. In addition, given that the associations between the parent-child INS and children’s behavioral indices were inconclusive, the real meaning of the parent-child INS to the children’s social development still needs more investigation.

Second, comparisons between mother, father or other caregivers (e.g., grandparents) are scarce. To date, most studies used mother-child dyads (Levy et al., 2017; Azhari et al., 2019, 2020; Miller et al., 2019; Nguyen et al., 2020a, 2021b,a; Quiñones-Camacho et al., 2020; Endevelt-Shapira et al., 2021; Zhao et al., 2021; Bembich et al., 2022; Reindl et al., 2022; Zivan et al., 2022), five studies used both mother-child and father-child dyads (Reindl et al., 2018; Wang et al., 2020; Kruppa et al., 2021; Deng et al., 2022a,b), two studies used father-child dyads (Azhari et al., 2021; Nguyen et al., 2021b), and no studies tested other caregiver-child dyads. The fact that most studies tested mother-child dyads makes sense as mothers are primary caregiver, but it has been shown, that the father plays important role in children’s development (Feldman, 2003; Liong, 2017), besides other caregivers such as grandparents also provide considerable help during the children’s growth (Attar-Schwartz et al., 2009; Mahne and Huxhold, 2015). The current explanations (i.e., attachment and social cognitive processes) regarding INS are not limited to mother-child relationships. For instance, father and other caregivers can also form a specific attachment relationship with the child which could result in specific INS with the child. Of note, the mother may induce very specific features compared with the father or other caregivers. For example, mothers and fathers co-create distinct types of behavioral synchrony with the infant; maternal synchrony is more cyclic and social oriented while paternal synchrony orients toward the environment and encourages exploration (Feldman, 2003). Recent findings have already shown a few different results between mother- and father-child dyads. For example, the mother-child INS decreased with the mother’s parenting stress (Azhari et al., 2019), but it is not the case for the father (Nguyen et al., 2021b). Nonetheless, there are also similar results for the mother-child and father-child dyads. For instance, the INS during cooperative puzzle-solving tasks was stronger than the independent task for both the mother-child (Nguyen et al., 2020b) and father-child (Nguyen et al., 2021b) dyads. Hence, future research would benefit from the use of systematic comparisons between the INS of the mother-, father-, and even other caregiver-child dyads as these would help to understand the importance of the different parenting roles on children’s social development.

Third, whether and how the parent-child INS changes with the children’s age were not tested. The children tested in existing studies were within four age groups, i.e., infants younger than 1 year old (Endevelt-Shapira et al., 2021; Nguyen et al., 2021a; Bembich et al., 2022), children about 2–5 years (Azhari et al., 2019, 2020, 2021; Nguyen et al., 2020a,b, 2021b; Quiñones-Camacho et al., 2020), children about 7–12 years (Levy et al., 2017; Reindl et al., 2018; Miller et al., 2019; Wang et al., 2020; Zhao et al., 2021; Zivan et al., 2022), and adolescents about 10–18 years (Deng et al., 2022a,b; Kruppa et al., 2021; Reindl et al., 2022). It is known that from infants to 5 years are preschool ages while 7–12 years are school ages and 10–18 years are in puberty. School education could also affect the social brain development of the children (see Lieberman, 2012 for a review) and the parent-child relationship changes a lot from childhood to puberty (Branje, 2018). Although parent-child behavioral synchrony affected the development of children’s social skills across childhood and up to adolescence (see Feldman, 2012 for a review), it is worth to test whether the parent-child INS would be different before and after the school age or before and after the purberty. In other words, whether the parent-child INS changes with the growth of the children needs to be answered in future research, which would provide a full view as to what extent the parent-child relationship affects the social development of the children.

Forth, two aspects of methodology pitfalls are worth noting. One is that the recording of brain activities using fNIRs only targets cerebral cortex regions (e.g., TPJ) of the social brain network. Whether other key nodes in the social brain network such as insular, medial prefrontal cortex, cingulate cortex and precuneus would show similar features of the parent-child INS are still unknown. The other pitfall is the inconsistency of the INS measurement. Currently, the INS measurement is far from established. For example, some studies used cross-correlation analysis (Azhari et al., 2020, 2021; Quiñones-Camacho et al., 2020), while other studies used coherence analysis (i.e., WTC) (Reindl et al., 2018; Miller et al., 2019; Nguyen et al., 2020a,b, 2021b; Wang et al., 2020; Zhao et al., 2021). The discrepancy in INS calculation has to be taken into account when comparing the results of different studies. In addition, most studies used time-aligned indices of parent-child INS (e.g., Levy et al., 2017; Azhari et al., 2019; Miller et al., 2019; Nguyen et al., 2020b,2021b) and only one study used the time-lagged index (Zhao et al., 2021). The pattern of time-lagged INS has been found between the leader and the follower (i.e., the neural activity of the follower was a few seconds lags behind that of the leader) during the leader guided cooperation (Sänger et al., 2013; Konvalinka et al., 2014; Jiang et al., 2015). This kind of leader-follower relationship during interaction was similar to the parent-child interaction in daily life, for example, the parents always needed to teach the children life skills or provide guidance when the children got stuck in difficult tasks. In these types of situations, it is possible that the parent-child INS would also show the time-lagged pattern. However, up to now, only one study used the parent-guided drawing task and the time-lagged parent-child INS as indices (Zhao et al., 2021), while the remaining studies used time-aligned INS indices (e.g., Levy et al., 2017; Azhari et al., 2019; Miller et al., 2019; Nguyen et al., 2020b,2021b). Future investigation should focus on the parent-guided interactions, whereas testing the time-lagged INS would provide new insights to understand the neural correlates of the parent-child interaction.

To summarize, existing studies have demonstrated the specificity of the parent-child INS, investigated the situational factors that affect the parent-child INS, and correlated the parent-child INS with the parents’ and child’s state-like and trait-like behavioral indices. The existing findings also indicate that the parent-child INS may provide a very promising avenue for parent-child relationship and the social development of the children. In spite of this, it is worth noting that these studies used different tasks, different gender of participants, and different indices, which constrained the comparisons between findings, and raised a number of questions to be answered by future studies.

## Author contributions

YiL and JL organized all the existing findings and wrote the manuscript. QW and YaL searched the literatures and revised the manuscript. All authors contributed to the article and approved the submitted version.
